# Clinical, radiological, and electroencephalographic features of HHV-6 encephalitis following hematopoietic stem cell transplantation

**DOI:** 10.1016/j.amsu.2020.10.022

**Published:** 2020-10-21

**Authors:** Ahmed Yassin, Abdel-Hameed Al-Mistarehi, Khalid El-Salem, Aiman Momani, Majdi Al Qawasmeh, Rafael Rodriguez, Sudhakar Tummala

**Affiliations:** a Assistant Professor of Neurology, Clinical Neurophysiology, Epilepsy, and Medical Quality, Division of Neurology, Department of Neurosciences, Faculty of Medicine, Jordan University of Science and Technology, Irbid, Jordan; b Department of Public Health and Family Medicine, Faculty of Medicine, Jordan University of Science and Technology, Irbid, Jordan; c Professor of Neurology, Division of Neurology, Department of Neurosciences, Faculty of Medicine, Jordan University of Science and Technology, Irbid, Jordan; d Division of Neurology, Department of Neurosciences, Faculty of Medicine, Jordan University of Science and Technology, Irbid, Jordan; e Assistant Professor of Neurology, Division of Neurology, Department of Neurosciences, Faculty of Medicine, Jordan University of Science and Technology, Irbid, Jordan; f Consultant Neurologist and Neurophysiologist, Neurophysiology Center, Professional Association, Tampa, FL, USA; g Division of Cancer Medicine, Department of Neuro-Oncology, The University of Texas MD Anderson Cancer Center, Houston, TX, USA

**Keywords:** Transplantation, Stem cells, Seizures, MRI, Encephalitis, Viral infection, HHV-6

## Abstract

**Background:**

To study the clinical, radiological, electroencephalographic, and cerebrospinal fluid (CSF) features of Human Herpes Virus 6 (HHV-6) encephalitis in leukemia patients underwent allogeneic hematopoietic stem cell transplantation (HSCT).

**Methods:**

We retrospectively reviewed all leukemia patients who underwent allogeneic HSCT between January 2010 and December 2018. The clinical, radiological, electroencephalographic, and CSF features of those with HHV6 encephalitis were recorded.

**Results:**

A total of five cases of HHV6 encephalitis were identified. Three patients had Chronic Myelogenous Leukemia, one had Chronic Lymphocytic Leukemia and one had Acute Lymphoblastic Leukemia. All of them presented a few months after transplantation with altered mental status. Comorbidities included pancytopenia, sepsis, graft versus host disease, and multi-organ failure. EEG showed focal seizures originating from temporal lobes in two patients, generalized or focal periodic discharges in three patients, focal slowing in two patients, and diffuse slowing in three patients. MRI brain showed T2/FLAIR hyper-intensities in four patients; two of them in bilateral temporal lobes, one in the thalamus/hypothalamus/brainstem/cerebellum/basal ganglia, and one in the periventricular areas. CSF showed pleocytosis, high protein, and positive HHV-6 PCR. Foscarnet was used as an anti-viral agent. Anti-epileptics used were phenytoin, levetiracetam, and valproic acid. Four patients died in a few months, whereas one recovered completely.

**Conclusions:**

HHV-6 encephalitis can add significant morbidity and mortality to leukemic patients following allogeneic HSCT. Patients present with typical clinical features of encephalitis. Salient EEG characteristics include periodic discharges or overt temporal lobe seizures. MRI findings are T2/FLAIR signal hyperintensities, mainly in the temporal lobes.

## Highlights

• HHV-6 encephalitis is a fatal complication of allogeneic hematopoietic stem cell transplantation (HSCT).
• Common features include confusion, fever, headache, and seizures.
• EEG can show focal or generalized periodic discharges or overt temporal lobe seizures.
• MRI may show temporal lobe hyperintensities and diffusion restriction.
• Starting Foscarnet and antiepileptic drugs as early as post HSCT patients develop confusion is recommended.

## 1 Introduction

Human Herpes Virus 6 (HHV-6) is a virus of the Roseolovirus genus in the Betaherpesvirnae subfamily [1]. Two species are recognized, HHV-6A and 6B. HHV-6-B encephalitis after bone marrow transplantation was first reported by Drobyski et al., in 1994 [2]. Reactivation of HHV-6 commonly occurs following allogeneic hematopoietic stem cell transplantation (HSCT) due to immunosuppression. A few case reports and small case series have described HHV-6 encephalitis as a complication in the post allogeneic HSCT course [3,4].

Common clinical features of HHV-6 encephalitis include altered level of consciousness, changes in personality and behavior, and seizures [5]. Foscarnet is the antiviral agent of choice according to guidelines [6].Given the mortality and morbidity associated with this infection and the vulnerability of patients who undergo allogeneic HSCT, early detection and treatment of this infection in this group of patients is of paramount importance.

Our study aims to retrospectively analyze the clinical, radiological, electroencephalographic, and cerebrospinal fluid (CSF) features of all patients who developed HHV-6 encephalitis after bone marrow transplantation for leukemia at MD Anderson Cancer Center (MDACC), Houston, Texas, within the period from 2010 to 2018.

## 2 Materials and methods

We conducted a single-center, retrospective cohort review of all patients with acute and chronic leukemia who underwent allogeneic HSCT at MDACC, Houston, Texas within the period from January 2010 to December 2018. The recipients received cyclosporine for the prevention of Graft versus Host Disease (GvHD). They were given prophylactic antimicrobials after allogeneic HSCT to prevent viral, bacterial, and fungal infections.

The presence of unexplained neuropsychological symptoms along with positive polymerase chain reaction (PCR) results for HHV-6 in CSF and the absence of other objective explanations of CNS dysfunction constituted the commonly used criteria to diagnose HHV-6 encephalitis [7]. HHV-6 positivity was defined as at least one positive HHV-6 PCR test (above the PCR test's threshold of detection) at any time following HSCT. Patients who had unexplained neuropsychological disorders without CSF data were excluded from this study.

Detailed Clinical, radiological, electroencephalographic, and CSF data were collected by reviewing medical records. All procedures performed in this study involving human participants were reviewed and ethically approved by the Institutional Review Board (IRB) and the research and ethics committee at MD Anderson Cancer Center, Houston, TX, USA. This study was conducted following the 1975 Helsinki declaration (including its later amendments). This work has been reported based on STROCSS 2019 guidelines [8], and the research protocol was registered in the Research Registry with the unique identification number of 5963.

## 3 Results

Five cases of HHV-6 encephalitis were found. Those included three patients with Chronic Myelogenous Leukemia (CML), one with Chronic Lymphocytic Leukemia (CLL), and one with Acute Lymphoblastic Leukemia (ALL). All patients developed altered mental status (AMS) within 1–3 months after transplantation. Other clinical symptoms/signs included seizures (in three out of five patients), headache (in three out of five patients), fever (in three out of five patients), and extrapyramidal features (in one out of five patients). Four patients required ICU admission with intubation. Comorbidities included pancytopenia, sepsis, GvHD, and multi-organ failure.

**Fig. 1:**
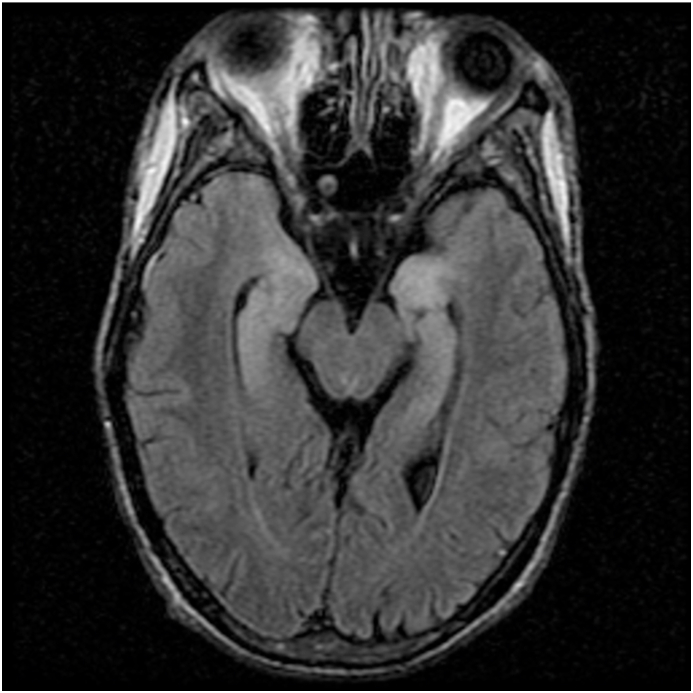
MRI-FLAIR of patient 2; showing hyperintensity of bilateral mesial temporal areas including unci/hippocampi. FLAIR: Fluid-Attenuated Inversion Recovery.

**Fig. 2:**
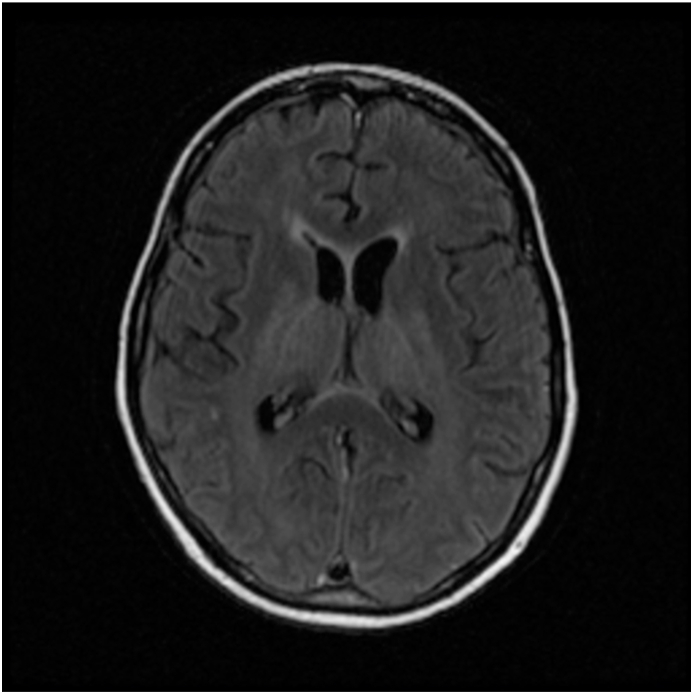
MRI-FLAIR of patient 3; showing hyperintensity in the thalami and the posterior limbs of the internal capsules bilaterally. FLAIR: Fluid-Attenuated Inversion Recovery.

**Fig. 3:**
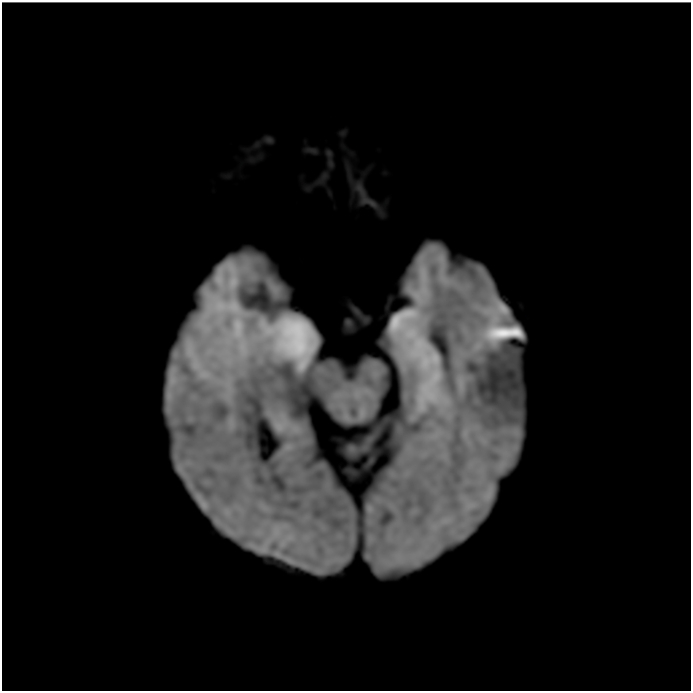
MRI-DWI of patient 5; showing restricted diffusion in right > left mesial temporal lobes. DWI: Diffusion-Weighted Imaging.

MRI brain showed T2/FLAIR hyper-intensities in four patients; two in bilateral temporal lobes with corresponding diffusion restriction, one in the thalamus/hypothalamus/brainstem/cerebellum/basal ganglia, and one in the periventricular areas. Fig. 1, Fig. 2, Fig. 3 show examples of MRI changes in patients 2, 3, and 5, respectively.

**Fig. 4:**
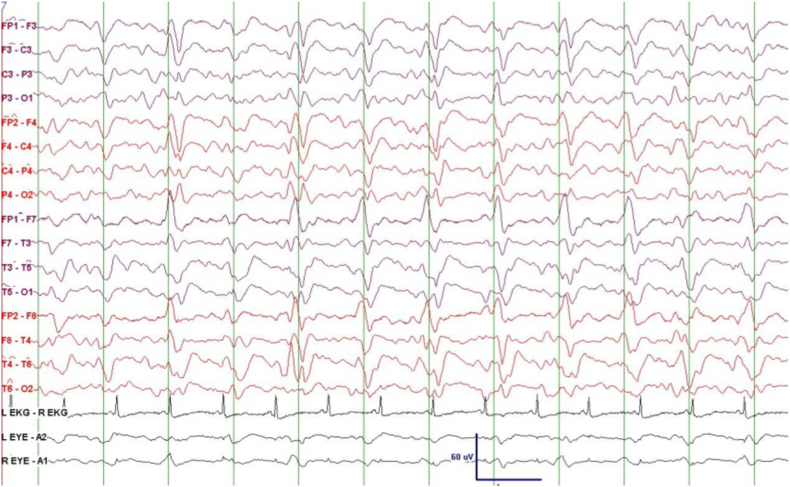
EEG epoch of patient 1; showing Generalized Periodic Discharges (GPDs) of 1 Hz frequency.

**Fig. 5:**
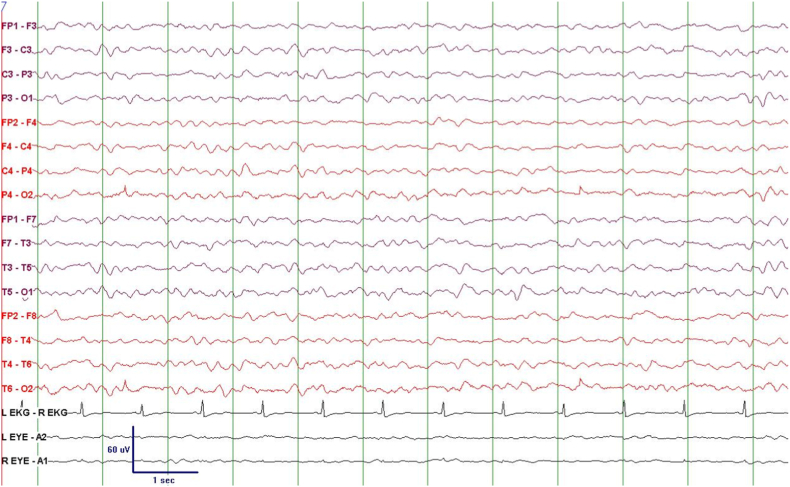
EEG epoch of patient 3, 14 days after improvement; showing generalized theta/delta slowing.

**Fig. 6:**
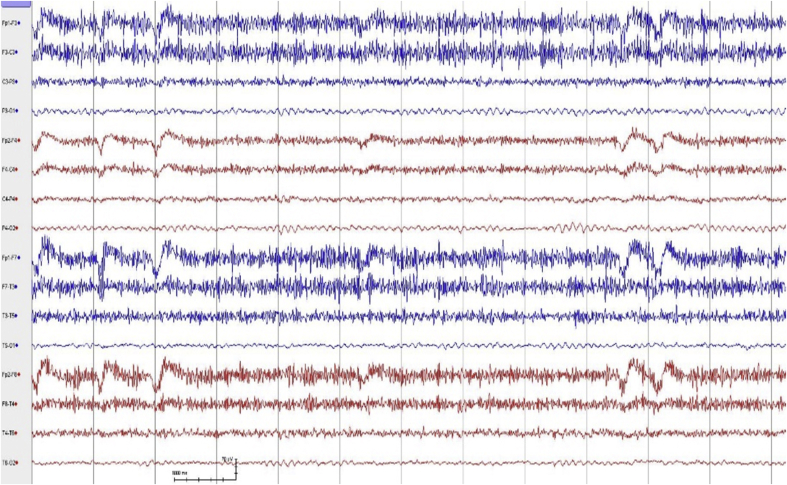
Normal awake EEG of patient 4 and following commands.

EEG showed focal electrographic seizures originating in the temporal lobes in two patients, generalized and focal periodic discharges in three patients, focal slowing in two patients, and diffuse slowing in three patients. Fig. 4, Fig. 5, Fig. 6 show examples of Electroencephalogram (EEG) findings in patients 1, 3, and 4, respectively.

The spinal fluid analysis showed pleocytosis and high protein. Spinal fluid HHV-6 PCR was +ve in all patients. Foscarnet was used as an anti-viral agent for all patients. Anti-epileptics used included phenytoin, levetiracetam, and valproic acid. Four patients died within a few months, two as a direct consequence of encephalitis and two due to other post-transplant complications. One patient had a complete recovery. Table 1 demonstrates all patients’ clinical, radiological, electroencephalographic, and CSF features.

**Table 1:** The clinical, radiologic, and electroencephalographic features and disease course including treatment and outcome for the patients.

Patient's number	Leukemia Type	Presentation	Intubation	MRI	EEG	CSF	Treatment	Outcome
**1**	CLL	AMS/coma	Yes	Periventricular FLAIR hyperintensities	Diffuse slowing and GPDs	WBC 101 Glucose 40 Protein 114 HHV-6 PCR + ve	Foscarnet, Phenytoin	Died after 3 months
**2**	CML	AMS, seizure	Yes	Bitemporal FLAIR hyperintensities and DWI restrictions	Seizures/focal slowing/periodic discharges from left temporal lobe	WBC 50 Glucose 50 Protein 80 HHV-6 PCR + ve	Foscarnet, Levetiracetam and phenytoin then Levetiracetam alone	Recovered and extubated but died after 4 months
**3**	ALL	AMS/Coma, seizure, headache, fever, extrapyramidal movements	Yes	Thalamic/Hypothalamic/brainstem/cerebellum/basal ganglia FLAIR hyperintensities	Diffuse slowing and seizures/focal slowing/periodic discharges from left temporal lobe	WBC 7 Glucose 47 Protein 71 HHV-6 PCR + ve	Foscarnet, Valproic Acid and Phenytoin then Valproic Acid and Levetiracetam	Died after 2 months
**4**	CML	AMS, headache, fever	NO	Normal	Normal	WBC 67 Glucose 70 Protein 173 HHV-6 PCR + ve	Foscarnet, NO antiepileptic drugs	Complete recovery. CSF cleared from HHV-6
**5**	CML	AMS, headache, fever, seizure	Yes	Bitemporal FLAIR hyperintensities and DWI restrictions	Diffuse slowing	WBC 75 Glucose 55 Protein 82 HHV-6 PCR +	Foscarnet, Phenytoin	Died of GvHD complications in 2 months

MRI: Magnetic Resonance Imaging; EEG: electroencephalogram; CSF: Cerebrospinal Fluid; CLL: Chronic Lymphocytic Leukemia; CML: Chronic Myelogenous Leukemia; ALL: Acute Lymphoblastic Leukemia; AMS: Altered Mental State; FLAIR: Fluid-Attenuated Inversion Recovery; DWI: Diffusion-Weighted Imaging; GPDs: Generalized Periodic Discharges; WBC: White Blood Cells Count; PCR: Polymerase Chain Reaction; GvHD: Graft-Versus-Host Disease.

One unique case was that of patient no. 3. This was a 52-year old lady with ALL who presented with altered mental status, headache, and fever. She underwent an umbilical cord blood transplant in the month before the presentation. Shortly after admission, she developed seizures, generalized tremulousness, cogwheel rigidity, random myoclonus, dysconjugate gaze, and asterixis. The patient deteriorated and went into a coma and was intubated. Analysis of her CSF confirmed infection by HHV-6. Her brain MRI showed abnormal T2 signal primarily involving the brainstem, hypothalamus, thalamus, left globus pallidus, and cerebellum **(**Fig. 2**)**. Her initial EEG showed an electrographic seizure originating in the left posterior temporal/occipital region, focal slowing, and periodic discharges in the left temporal lobe. The patient was treated with Foscarnet, Valproic acid, Phenytoin, and Levetiracetam. Her EEG, 14 days after improvement, revealed no seizures or periodic discharges with improvement in the background which showed generalized theta/delta slowing (Fig. 5). Unfortunately, the patient developed renal and respiratory failure and expired two months after the onset of encephalitis. Postmortem pathological dissection of the brain showed marked astrogliosis, microgliosis, demyelination, and neuronal loss throughout the nervous system. Viral culture from the right uncus did not reveal viral particles. Fungal and bacterial cultures showed no growth.

## 4 Discussion

HHV-6 encephalitis appears to be a major and potentially fatal complication of allogeneic HSCT. AMS appears to be the most common initial presentation. This symptom occurred in our cohort of patients within 1–3 months of transplantation. Other common clinical symptoms/signs included seizures, headaches, and fever. This is consistent with the clinical features described in other published studies [[3], [4], [5]]. The rapid deterioration of our patients' health status was accompanied by the failure of other systems (hematopoietic, respiratory, and renal systems), sepsis, and development of GvHD. Four of our patients required ICU admission and intubation, which indicates the severity of their encephalitis and the comorbid conditions.

The findings of the MRI brain in our cohort of patients were T2/FLAIR and DWI changes mainly seen in the temporal lobes and limbic areas, as frequently seen with other viral encephalitides particularly herpes simplex encephalitis [9]. These findings are consistent with the findings of other published reviews on HHV-6 encephalitis [5,10].

Spinal fluid showed evidence of inflammation including pleocytosis and high protein. HHV-6 PCR was +ve in all patients, which is considered diagnostic of HHV-6 [11]. These findings are consistent with the findings of other studies [3].

EEG showed focal electrographic seizures coming from temporal lobes, focal and generalized periodic discharges, and focal and diffuse slowing, and these were also consistent with the findings of previously published studies [3,12]. Focal or lateralized periodic discharges that were seen in 2 of our patients are similar to the commonly described Lateralized Periodic Discharges (LPDs) seen with the more frequently encountered herpes simplex encephalitis [13].

Foscarnet was used to treat the infection in all of our patients. Foscarnet is the recommended antiviral agent according to guidelines [6] and has previously shown success in published cases [14].

Several limitations should be reported in this study. First, it has a small number of cases identified and analyzed which is the major limitation of our study. This is due to the low frequency of such complications in a similar setting. However, we believe knowledge about these cases can be extrapolated and used to predict similar case scenarios under similar conditions. Second, there was no characterization of HHV-6 into type A versus type B. Third, the study lacked data regarding HHV-6 status before transplantation as well as HHV-6 serum antibodies at the time of HHV6 diagnosis. This is of particular importance to know whether HHV6 encephalitis was a primary infection or a reactivation of a latent infection that was present before HSCT.

## 5 Conclusion

HHV-6 encephalitis can add significant morbidity and mortality to leukemic patients who undergo allogeneic hematopoietic stem cell transplantation. The clinical features are typical for encephalitis. EEG characteristics include focal and generalized periodic discharges and overt temporal lobe seizures. MRI findings include T2/FLAIR signal hyper-intensities primarily in the temporal lobes. We suggest that leukemic patients with altered mental status following transplant be empirically started on Foscarnet along with indicated antiepileptic agents until the results of spinal fluid PCR studies are obtained.

## Funding

No Funding was received for this study.

## Provenance and peer review

Not commissioned, externally peer reviewed.

## Compliance with ethical standards

All procedures performed in this study involving human participants were reviewed and ethically approved by the Institutional Review Board (IRB) of MD Anderson Cancer Center, Houston, TX, USA. This study was conducted in accordance with the 1964 Helsinki declaration and its later amendments or comparable ethical standards.

## Availability of data and materials

The datasets generated and analyzed during the current study are available with the corresponding author.

## Authors' contributions

All authors have seen and approved the content, fulfill the criteria for the Authorship, and have contributed significantly to the work. All authors presented substantial contributions to the article and participated of correction and final approval of the version to be submitted.

## Declaration of competing interest

The authors declare that they have no competing interests.
